# 

**Published:** 1885-07

**Authors:** 


					﻿COLLABORATORS.
CHICAGO :
Professor J. A. ALLEN, M. D., LL. D.
Professor E. ANDREWS, M. D., LL. D.
Professor W. H. BYFORD, A. M., M. D.
Professor O. C. DeWOLF, A. M., M. D.
Professor E. C. DUDLEY, A. B., M. D.
Professor J. H. ETHERIDGE, A. M., M. D.
Professor C. FENGER, M. D. .
Professor J. N. HYDE, A. M., M. D.
Professor E. F. INGALS, A. M., M.D.
Professor H. A. JOHNSON, M. D., LL. D.
CHARLES GILMAN SMITH. A - M„ M.D
J. F. TODD, M. D.
MILWAUKEE, WISCONSIN:
Professor N. SENN. M, D.
WASHINGTON, D. C:
S. O. RICHEY, M. D.
UNITED STATES ARMY:
SURGEON, D. L. HUNTINGTON, M. D.
UNITED STATES NAVY:
MEDICAL DIRECTOR, J. M. BROWNE, M. D
MEDICAL DIRECTOR, A L. GIHON, A. M.. M. >
SUBSCRIPTION PRICE, $3.00 PER YEAR.
COLLABORATORS.
CHICAGO :
Professor J. A. ALLEN, M. D., LL. D.
Professor E. ANDREWS, M. D., LL. D.
Professor W. H. BYFORD, A. M., M. D.
Professor O. C. DeWOLF, A. M., M. D.
Professor E. C. DUDLEY, A. B., M. D.
Professor J. H. ETHERIDGE, A. M., M. D.
Professor C. FENGER, M. D.
Professor J. N. HYDE, A. M., M. D.
Professor E. FLETCHER INGALS, M.D.
Professor H. A. JOHNSON, M. D., LL. D.
CHARLES GILMAN SMITH, A. M., M.D
J. F. TODD, M. D.
MILWAUKEE, WISCONSIN:
Professor N. SENN, M, D.
WASHINGTON, D. C :
S. O. RICHEY, M. D.
UNITED STATES NAVY :
MEDICAL DIRECTOR J. M. BROWNE, M. I).
				

